# Implementation of adaptive dose-finding designs in two early phase haematological trials: clinical, operational, and methodological challenges

**DOI:** 10.1186/1745-6215-14-S1-O75

**Published:** 2013-11-29

**Authors:** Christina Yap, Charlie Craddock, Graham Collins, Josephine Khan, Shamyla Siddique, Lucinda Billingham

**Affiliations:** 1 MRC Midland Hub for Trials Methodology Research, University of Birmingham, Birmingham, UK; 2 Centre for Clinical Haematology, Queen Elizabeth Hospital, Birmingham, UK; 3 Oxford Cancer and Haematology Centre, Oxford University Hospitals NHS Trust, Oxford, UK; 4 Cancer Research UK Clinical Trials Unit, University of Birmingham, Birmingham, UK

## 

The majority of Phase I dose-finding trials in oncology have been dominated by the traditional up-and-down designs, such as 3+3 designs. However, in recent years, there is an emerging interest in more innovative model-based dose finding methods such as the Continual Reassessment Method (CRM), though examples of implementing such adaptive designs in the medical literature remain limited. In this paper, we discuss three main challenges faced in the implementation of CRM in a Phase I trial in Acute Myeloid Leukaemia and a Phase I/II trial in T-cell Lymphoma, and possible solutions.

Despite the mounting evidence of the CRM's superior performance in identifying the maximum tolerated dose, it remains a challenge to communicate to clinicians the rationale for using a method with such a greater level of statistical complexity. Operational challenges include trialists' impression that dose-recommendations come from a "black-box", which contrast unfavourably with the transparent simple rules of a 3+3 design; and concerns that possible delays that might result. Methodological challenges include the different variants of a CRM design that could be considered, both from Bayesian and Maximum Likelihood frameworks, and how to choose an appropriate design that could be adapted to specific requirements of a trial.

We discuss proposed solutions to challenges encountered and introduce the use of Dose Transition Sample Paths, demonstrating how this can be used to aid understanding in the working of such adaptive designs. We will show that it is a valuable planning tool to achieve a seamless, clear transition for each dose-recommendation.

